# Diaphragmatic Pleurisy

**Published:** 1907-05-11

**Authors:** J. A. Nixon

**Affiliations:** Assistant Physician to the Bristol Royal Infirmary


### DIAPHRAGMATIC PLEURISY.

By J. A. NIXON, M.B., M.R.C.P., Assistant Physician to the Bristol Royal Infirmary.

The symptoms of pleurisy affecting the diaphragm alone without extending to the adjacent pleura or lung, although not constituting a separate disease clinically or pathologically, are yet so misleading and of such importance that they warrant a separate consideration. This the German authorities refuse to recognise; and without denying the attitude adopted in Germany to be perfectly correct and agreeing with the contention that diaphragmatic pleurisy has no place in a pathological classification of diseases, still the localisation of the inflammatory process is of such importance to the patient by reason of the difficulties of differentiating this condition from some abdominal inflammation, that, to the physician or surgeon who is called upon to act promptly rather than to discuss subtly, a knowledge of the salient points in which this condition is at variance with the other forms of pleurisy is of the utmost importance.

With the single exception that diaphragmatic pleurisy may originate by extension from an inflammation of the peritoneal aspect, the etiology is that of any other form of pleurisy; whether the condition is rarer or not than that of other forms is here an unprofitable discussion. The occasions on which it will be found to present its characteristic and puzzling features are certainly not frequently met with.

In former times, when the only difference between peritonitis and pleurisy lay in the fact that in the former disease the patient would probably die in spite of bleedings and calomel, while in the latter the same treatment need not necessarily prove fatal, to delay the diagnosis to the third or later day from the onset was not of vital moment either to the physician, the patient, or friends.

Recent advances in abdominal surgery have, however, made an early diagnosis of "the abdominal catastrophe" so urgent that to have a patient die of peritonitis unoperated on is an offence so dire that surgery awards it the first place in the catalogue of crime; equally sincerely, but less vehemently, does the patient deprecate an exploratory abdominal section as the prelude to a severe attack of lobar pneumonia. How then, can one avoid the silent reproach of an autopsy preventable by timely operation, or the eloquence of a convalescent who thinks he has been looked inside of—for practice?

First and foremost: the possibility of a pleurisy localised to the diaphragm must be recognised; a diagnosis which must be arrived at immediately, within an hour of the onset of symptoms, cannot be made by mother wit alone or by a long train of inductive reasoning. A knowledge of the actual happenings is required; experience goes far to the avoiding of error, and not personal experience alone; for the occurrence is so comparatively rare that for a man to say he has never seen diaphragmatic pleurisy operated upon as a ruptured gastric ulcer is not to prove he may not do so in this instance. Latham's advice remains, like most of his precepts in medicine, sound to the present day: "Let a man use his own experience as best he can for the present; but let him not, on the strength of it, rebuke the experience of all past times, and dictate to the experience of all future; for, if he live long enough, nothing is more likely than that he may find himself fallen under his own reproof and inconveniently confronted by his own maxims."

A surgeon was sent for to see a supposed case of perforated gastric ulcer three times in twenty-four hours. On arriving the third time he was not annoyed; he declined to operate, but added: "If you have never seen a case of diaphragmatic pleurisy you could not help sending for me." In that case the pleural effusion appeared at the end of a week. Perhaps he had already operated on one such case.

A good rule in any doubtful case is to examine the bases of the lungs before operating.

Even before the time of Laennec, Andral had observed that diaphragmatic pleurisy might be mistaken for abdominal inflammation; and the remark has been quoted, sometimes acknowledging the source, more often as an original observation, in text-books and monographs ever since. The application of the observation was perhaps never fully insisted on until a year or two ago, when Barnard discussed the thoracic conditions simulating abdominal catastrophes.

### Symptoms.

The symptoms of acute diaphragmatic pleurisy are, as a rule, ushered in by severe abdominal pain, a rigor maybe, and a rise of temperature to 103° or 104°; the pulse-rate increases considerably; the abdomen is held rigid from the outset, is always tender to pressure; the knees are sometimes drawn up. Hiccough is a very constant symptom, and an early one; nausea and vomiting may be present. The expression becomes anxious, and there is the "fear of death" as in pericarditis; there may be the so-called "typical abdominal facies." Constipation is the rule.

The situation of the pain may suggest several other possible diagnoses. Fenwick mentions four cases imitating respectively gall-stone colic, perityphlitis, acute perforative appendicitis, and general peritonitis; it has affected the loins and given rise to a supposed lumbago. Perforated gastric ulcer is perhaps the most probable suspicion that diaphragmatic pleurisy will arouse, while subphrenic abscess and inflammation of the spleen have on occasions been closely simulated. I have seen perforation in typhoid fever most remarkably imitated, and only finally disproved by the appearance of a pleural effusion and the favourable course of the malady.

### Diagnosis.

Having regard, then, to the necessity of diagnosing *something* when there is the possibility of an abdominal catastrophe having occurred, not justified in waiting twenty-four hours to form an opinion of any sort, but compelled to make on the spot the choice between operation and expectant treatment, what are the important points in "happily guessing," as John Brown put it, at the real state of affairs?

The prominent symptoms—the pain, rigidity, and tenderness of the abdomen, the facial expression, the knees drawn up, the high temperature, rapid pulse, vomiting, and constipation—will all suggest a possibility of peritonitis, which the persistent hiccough—so prominent a symptom—will go far to clinch. Yet to the practised observer there is usually some symptom which fits in but ill with this diagnosis.

The respiration, the catchy half-drawn inspiration of pneumonia, the hand pressed to the chest to check the full expansion of the thorax, in some cases actual orthopnœa, will form a slender indication that there is something not wholly consistent with peritonitis; the patient lies still, not writhing with the free movements of a renal colic.

The pulse is full and soft, not thready as in peritonitis, nor irregular as in pericarditis.

The tenderness is not limited to the abdomen, nor merely elicited on deep pressure, but is superficial, is referred to points along the course of the phrenic nerve, to the attachment of the sterno-mastoid muscle, to the interspaces close to the sternal margin, to the costo-diaphragmatic attachment, and may be brought out by pressure on the false ribs. There is a special point of tenderness, even if spontaneous pain is not complained of, formed by the intersection of two lines—the one prolonged downwards from the sternal border, the other a prolongation forwards from the osseous part of the tenth rib.

The abdomen is retracted, not distended, save in children who distend rapidly with pneumonia or any interference with diaphragmatic movement.

Delirium occurs early, and is a prominent feature, unlike peritonitis, where, at the outset, the full faculties are preserved. On auscultation the bases of the lungs behind may show a deficiency of expansion not usual in any intra-abdominal inflammation or colic; a sticky crepitation may be caught, no more, but enough to make the hearer pause in his ready diagnosis.

And having paused to think, is there any further help to be obtained from examination, or is an exploratory operation to be advised?

There is one method of diagnosis still at hand which may render the most valuable assistance.

In pleurisy, whether of tuberculous or pneumococcic origin, leucocytosis is not common at the actual time of onset; in any perforative peritonitis it occurs early and is very marked. Probably it would not be safe to rely on this single sign any more than upon another occurring alone. I have seen leucocytosis of a moderate degree where a perforation was suspected in typhoid fever, but did not exist; yet there is no doubt that a marked leucocytosis may be expected at an early stage in any abdominal catastrophe, while in the case of diaphragmatic pleurisy it would be unusual to find it coinciding with a sudden onset.

To recapitulate. The differential diagnosis between diaphragmatic pleurisy and intra-peritoneal lesions will be found to lie in the character of the respirations: the full, soft pulse; the distribution of tenderness and the localising points; retraction rather than distension of the abdomen; early manifestation of delirium; the blood-count.

The task of distinguishing this form of pleurisy from pericarditis is on occasions almost impossible; the lapse of time alone will make the difference clear.

In fact, in all other cases of doubt, except when there is the possibility of a grave abdominal condition, it is safe to predict that the course of diaphragmatic pleurisy will be such that by the third or fifth day at latest there will be some extension of the inflammation to the surrounding parts which will clear away all difficulties in diagnosis.

Adults will rarely die in the early stages, though in children a rapid—almost sudden—fatal termination is by no means uncommon, and the cause of death is usually only ascertained at the autopsy.

